# Principles of Human Physiology, with Their Chief Applications to Pathology, Hygiene, and Forensic Medicine

**Published:** 1845-01

**Authors:** 


Art. VII.—*Principles of Human Physiology, with their chief applica tions to Pathology, Hygiene, and Forensic Medicine. Specially designed for the use of Students.* By W. B. Carpenter, m.d. f.r.s., Fullerian Professsor of Physiology in the Royal Institution of Great Britain. Second Edition.—*London*, 1844. 8vo, pp. 746.

This volume reached us at so late a period of our quarterly labours, that we are able to do little more, at present, than to announce its publication. It is, however, though only a second edition, much too important a work to be so disposed of; we, therefore, purpose to notice it more formally in our next Number, more especially as regards the improvements and additions contained in it. Both of these are numerous and important; and, as the profession has, moreover, sanctioned our former judgment by so soon calling for a republication of the work, we feel more than justified in here repeating what we said in our review of the first edition :—"Our author has provided us with a work admirably calculated not only to guide and direct the student of physiology, but, from the agreeable mode in which old facts are presented, and new views opened up, also to afford pleasure and instruction to the deeply-learned in this branch of medical science. The style is everywhere easy, perspicuous, and appropriate to the subject. The numerous woodcuts and engravings, [greatly increased in number in this edition,] with which the descriptions are illustrated, are judiciously selected, and excellently executed. We speak advisedly when we say, that we know of no work on physiology from which the student is likely to derive so much advantage; the whole of it reflects the highest honour upon the talents, knowledge, and judgment of the author." (Brit. and For. Med. Rev., April 1842.)

